# Polymorphisms in the CHIT1 gene: Associations with colorectal cancer

**DOI:** 10.18632/oncotarget.9138

**Published:** 2016-05-02

**Authors:** Fei-Feng Li, Peng Yan, Zhi-Xun Zhao, Zheng Liu, Da-Wei Song, Xing-Wang Zhao, Xi-Shan Wang, Gui-Yu Wang, Shu-Lin Liu

**Affiliations:** ^1^ Genomics Research Center, State-Province Key Laboratory of Biopharmaceutical Engineering, Harbin Medical University, Harbin, China; ^2^ Translational Medicine Research and Cooperation Center of Northern China, Heilongjiang Academy of Medical Sciences, Heilongjiang, China; ^3^ Department of Colorectal Surgery, Second Affiliated Hospital of Harbin Medical University, Harbin, China; ^4^ Department of Colorectal Surgery, Cancer Institute and Hospital, Chinese Academy of Medical Sciences and Peking Union Medical College, Beijing, China; ^5^ Department of Microbiology, Immunology and Infectious Diseases, University of Calgary, Calgary, Canada

**Keywords:** colorectal cancer, CHIT1, C-reaction protein, gene expression, microbe

## Abstract

Colorectal cancer (CRC) is one of the most common solid tumors worldwide, often associated with inflammation. The microbes in the human intestine have a key role in inflammations and CRC. Chitotriose renders growth advantage to some bacteria, especially some pathogens, and thus has a role in inflammations. The enzyme chitotriosidase, encoded by the *CHIT1* gene of the host, may degrade chitotriose with different efficiencies depending on the alleles. We sequenced the *CHIT1* gene for 320 Chinese Han CRC patients and 404 normal controls, and focused on variations rs61745299 and rs35920428 within the *CHIT1* gene for their possible roles in CRC. Statistical analyses were conducted using Chi-Square Tests as implemented in SPSS (version 19.0). Multiple sequence alignment was conducted using the Vector NTI, and protein expression levels were analyzed by western blotting. The two variations, rs61745299 and rs35920428 within the CDS region of *CHIT1* gene, were associated with the risk of CRC (both with *P* values < 0.001). Western blotting analysis showed that the variations increased the expression levels of the *CHIT1* and C-reaction protein genes in the cancer tissue. We conclude that the two variations of *CHIT1*, rs61745299 and rs35920428, increase expression of the gene and are associated with CRC in Chinese Han populations.

## INTRODUCTION

Colorectal cancer (CRC) is one of the most common types of solid tumors worldwide [1]. Annually, over 96,000 new cases of colon cancer and 40,000 new cases of rectal cancer occur in the USA [2]. Most CRC cases are sporadic, with 15–25% of the cases having a family history [3] and 5% diagnosed cases having inherited CRC syndrome [4]. Several genes have been identified for their involvement in CRC [5]; however the precise etiologic factors of CRC are essentially unclear.

In addition to genetic predisposition, contribution of the intestinal microbiota to the development of CRC is gaining attention. Some intestinal bacteria may induce inflammation and damage immune cells in the intestinal lamina propria [6]. When the composition of the normal microbial community is disturbed or shifted, microbial dysbiosis may occur, facilitating inflammatory processes [7] and increasing the risk of CRC [8]. The intestinal barrier dysfunction during inflammation may result in adenoma invasion by microbial products, which may induce inflammatory cytokines and facilitate tumor growth [9]. In the inflammation processes, interactions between the intestinal microbiota and the immune system increase the risk of CRC [9, 10]. Microbial dysbiosis also promotes inflammation and tumorigenesis, and intestinal inflammation in turn modifies intestinal microbial community and promotes tumor growth [10]. However, it is unclear whether the inflammatory process or CRC is caused by dysbiosis, or dysbiosis occurs as a consequence of inflammation.

Bacterial cell wall contains chitotriose, which may render many pathogenic bacteria selective growth advantages in highly competitive ecological niches [11, 12]. Chitotriose can protect bacteria from bactericidal actions [13, 14].

The enzyme chitotriosidase that degrades chitotriose is encoded by the *CHIT1* gene (chitinase 1, Gene ID: 1118) located on chromosome 1q32.1. Chitotriosidase is produced by human mature monocyte-derived macrophages and other tissue macrophages at the late stage of differentiation [15, 16]. It is a highly conserved enzyme, which promotes innate immunity and degrades chitotriose-containing pathogens [17, 18].

In order to elucidate the possible associations of *CHIT1* gene and the pathogenicity of CRC, we analyzed the transcribed regions and splicing sites of the gene and compared the gene sequences between 320 Chinese Han CRC patients and 404 normal controls. We found that two variations rs61745299 and rs35920428 within the CDS region of *CHIT1* gene were associated with the risk of CRC in the Chinese Han Population, and the variations increased expression levels of the *CHIT1* and *C-reaction protein* genes in the cancer tissue.

## RESULTS

### Clinical data

The clinical diagnosis was confirmed by three specialists in colorectal cancer in the Second Affiliated Hospital of Harbin Medical University. There was no history of other systemic abnormalities in these CRC patients and no previous tumor or familial history. All the CRC patients (*n* = 320, male 196, female 124, the min and max age were 16 and 87 respectively, and the average age was 59.27 years) and unrelated controls (*n* = 404, male 251, female 153, the min and max age were 50 and 70 respectively, and the average age was 58.69 years) were recruited for this study, and there were no statistical differences in the gender composition or age between the two groups (Table 1).

**Table 1:** Clinical characteristics of study population

Parameter	CRC	Control	F	t	*P*	95% CI	
						Up	Low
**Sample (*n*)**	320	404	-	-	-	-	-
**Male/Female (*n*)**	196/124	251/153	-	-	0.809	-	-
**Age (years)**	59.27 ± 12.46	58.69 ± 4.15	198.866	−0.792	0.429	−2.04739	0.87167

Data are shown as mean ± SD; between the two groups, there were no statistical differences of the age and gender composition.

### *CHIT1* gene analyses

**Figure 1:**
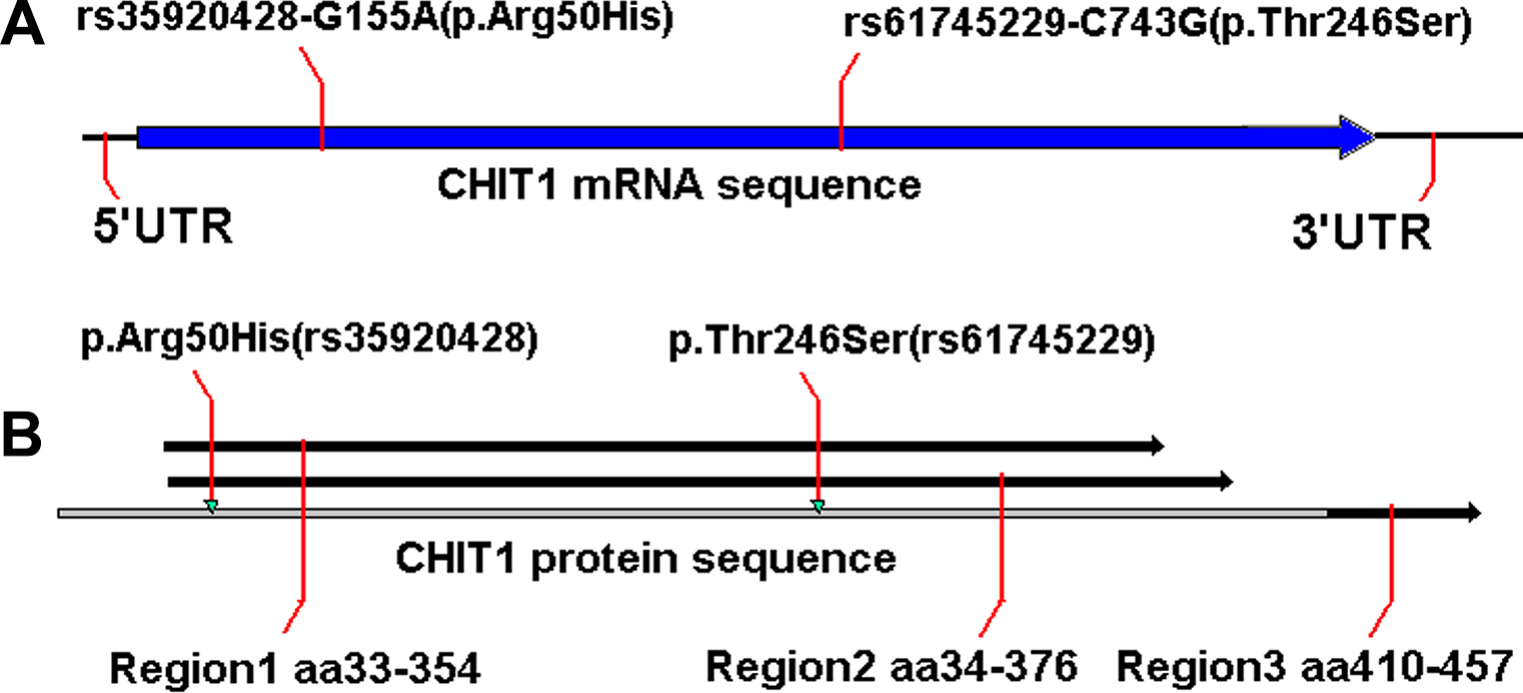
Schematic diagrams of the variations (**A**) Schematic diagrams of rs61745299 and rs35920428 locations in the *CHIT1* gene CDS region; (**B**) Schematic diagrams of p.Arg50His (rs61745299) and p.Thr246Ser (rs35920428) locations in the CHIT1 protein region 1 and region 2.

**Figure 2:**
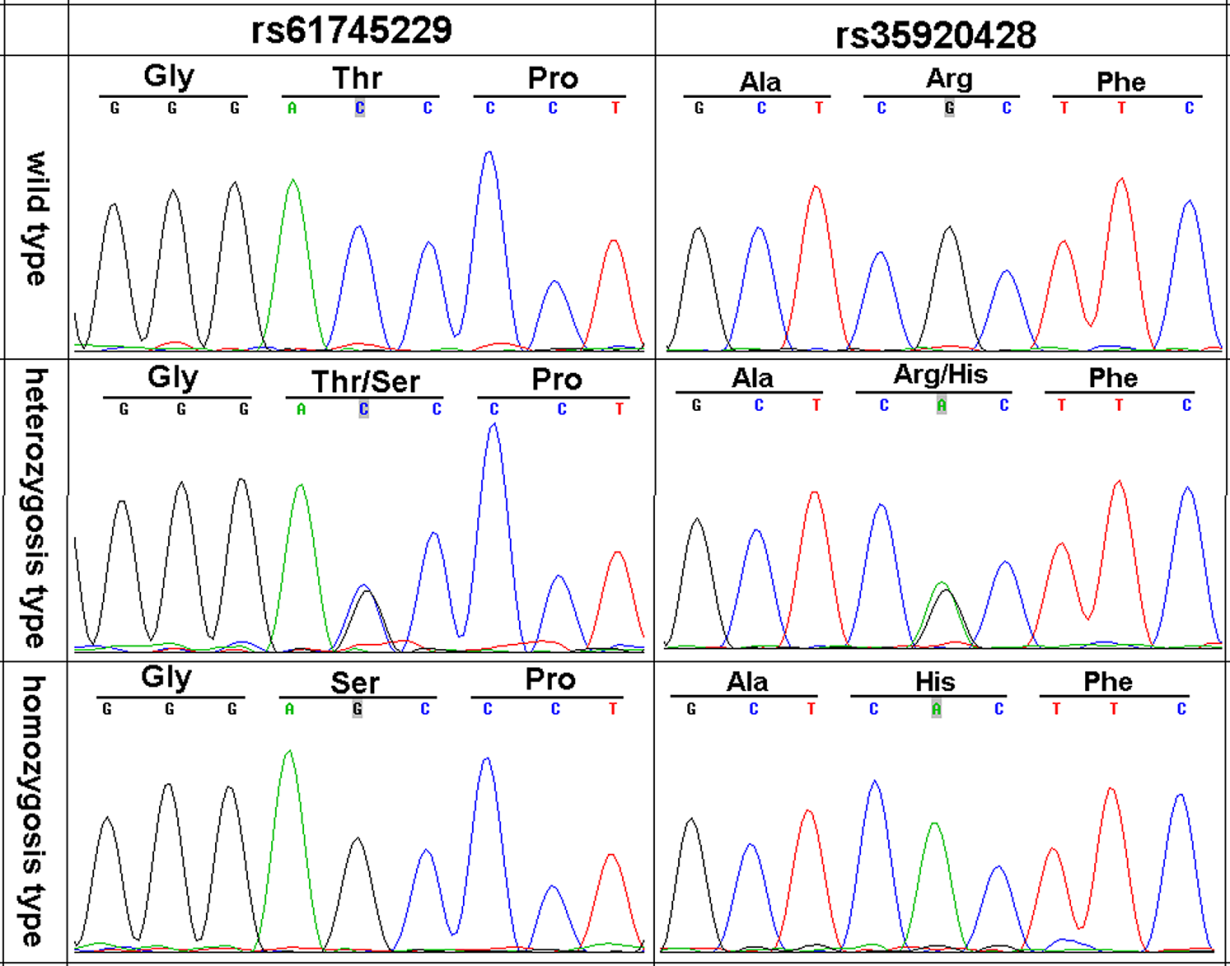
Three genotypes of DNA sequence chromatogram of rs61745299 and rs35920428 in *CHIT1* gene

We sequenced the *CHIT1* gene to test the hypothesis that germline common genetic variants in *CHIT1* may confer the susceptibility to CRC. We compared the transcribed regions and splicing sites of *CHIT1* and found the variants rs61745299 and rs35920428 within translated region (Figure 1A) and conserved domain family (Figure 1B). The conserved domain family included a large number of catalytically inactive chitinase-like lectins (chitolectins) such as YKL-39, YKL-40 (HCGP39), YM1, oviductin, and AMCase (acidic mammalian chitinase), as well as catalytically active chitotriosidases (CDD:119351). The genetic heterozygosity of the two variations was very high (Figure 2).

### Statistics analyses of polymorphism-disease association

To test any possible associations between *CHIT1* and CRC, we conducted SNP analyses and found that the variants rs61745299 and rs35920428 in the *CHIT1* gene were associated with the risk of CRC in the Chinese Han population (Tables 2, 3). At the same time, we also conducted the Hardy-Weinberg equilibrium test for the CRC and controls and it was in line with equilibrium.

**Table 2:** The genotype and allele frequency of rs61745299 and rs35920428 variations in 320 Chinese han sporadic colorectal cancer patients and 404 non-CRC controls

Variations	Group		Genotype frequency (%)			Allele frequency (%)	
**rs61745299**	Genotype		C/C	C/G	G/G	C	G
	CRC	320	253 (79.1)	66 (20.6)	1 (0.3)	572 (89.4)	68 (10.6)
	Controls	404	361 (89.4)	43 (10.6)	0 (0)	765 (94.7)	43 (5.3)
**rs35920428**	Genotype		G/G	G/A	A/A	G	A
	CRC	320	254 (79.4)	65 (20.3)	1 (0.3)	573 (89.5)	67 (10.5)
	Controls	404	361 (89.4)	42 (10.4)	1 (0.2)	764 (94.6)	44 (5.4)

SAP: sporadic adenomatous polyposis.

**Table 3:** rs61745299 and rs35920428 variations within *CHIT1* gene associated with risk of sporadic colorectal cancer in chinese populations

Variations	Type	Pearson Chi-square				Pearson's R			
		Value	Min count^a^	df	Asymp. Sig. (2-sided)	Value	Asymp. Std. error^b^	Approx. T^c^	Approx. Sig
**rs61745299**	Genotype	15.310^a^	0.44	2	**0.000**	−0.145	0.037	−3.929	**0.000^d^**
	Allele	14.190^a^	49.06	1	**0.000**	−0.099	0.026	−3.783	**0.000^d^**
**rs35920428**	Genotype	14.003^a^	0.88	2	**0.001**	−0.136	0.037	−3.678	**0.000^d^**
	Allele	12.731^a^	49.06	1	**0.000**	−0.094	0.026	−3.581	**0.000^d^**

a The minimum expected count;

b Not assuming the null hypothesis;

c Using the asymptotic standard error assuming the null hypothesis;

d Based on normal approximation.

### Conservation of the protein in evolution

**Figure 3:**
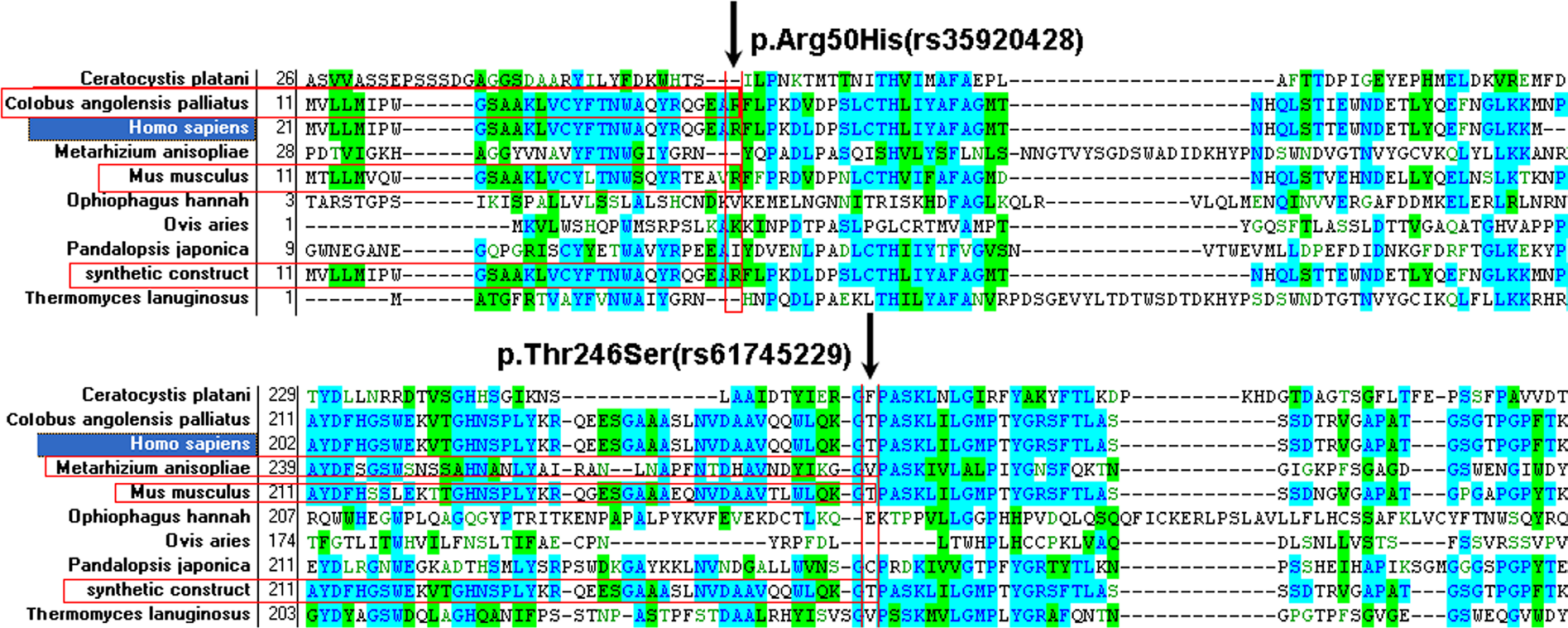
Conservation analysis of the CHIT1 protein sequences (**A**) multiple-sequence alignment of p.Arg50His (rs61745299); (**B**) multiple-sequence alignment of p.Thr246Ser (rs35920428). The conservation of the two residues was not high.

We compared the CHIT1 protein sequences from different species including birds, fishes, rodents and primates. Multiple-sequence alignment analysis showed that the conservation of all the CHIT1 protein sequences were very low and conservation of the 50 Arg and 246 Thr residues (SNPs position) were also very low (Figure 3).

### Expression levels of the wild and variant types of the *CHIT1* gene

**Figure 4:**
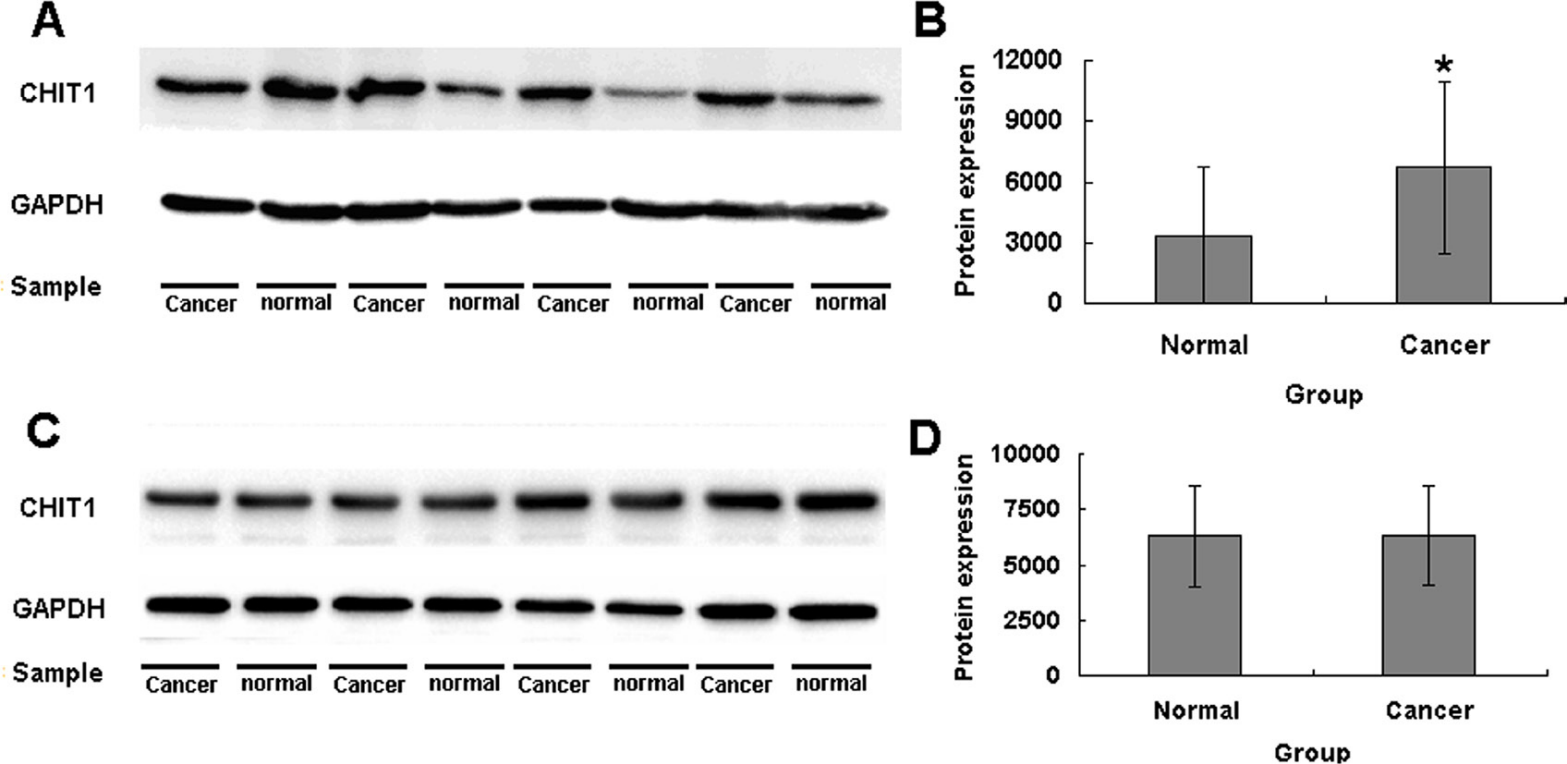
The expression levels of *CHIT1* in CRC patients (**A**) The expression levels of *CHIT1* in cancer tissues in the variations heterozygous and homozygous mutation type groups were higher than normal tissues. (**B**) There has a statistical difference between the cancer and normal tissue in the heterozygous and homozygous mutation type groups. (**C**) The expression levels of *CHIT1* in cancer tissues in the variations wild type group. (**D**) There has no difference of the expression level between the cancer and normal tissue in the variations wild type groups. The protein expression levels were normalized to GADPH.

**Figure 5:**
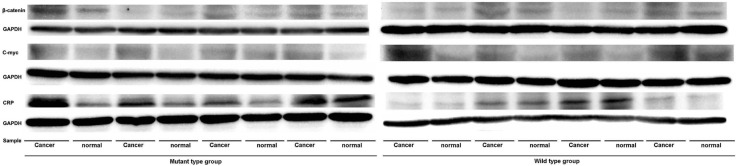
The expression levels of *C reaction protein, β-catenin, C-myc* genes of CRC patients The expression levels of the *C reaction protein* gene in the cancer tissues were higher than the normal tissue in the mutant type groups, however the wild type group was not. The expression levels of the *β-catenin* gene in the cancer tissues were higher than normal tissue in the mutant and wild type groups, however it was not obvious. The expression levels of the *C-myc* gene in the cancer tissues were higher than the normal tissue in the mutant and wild type groups.

**Figure 6:**
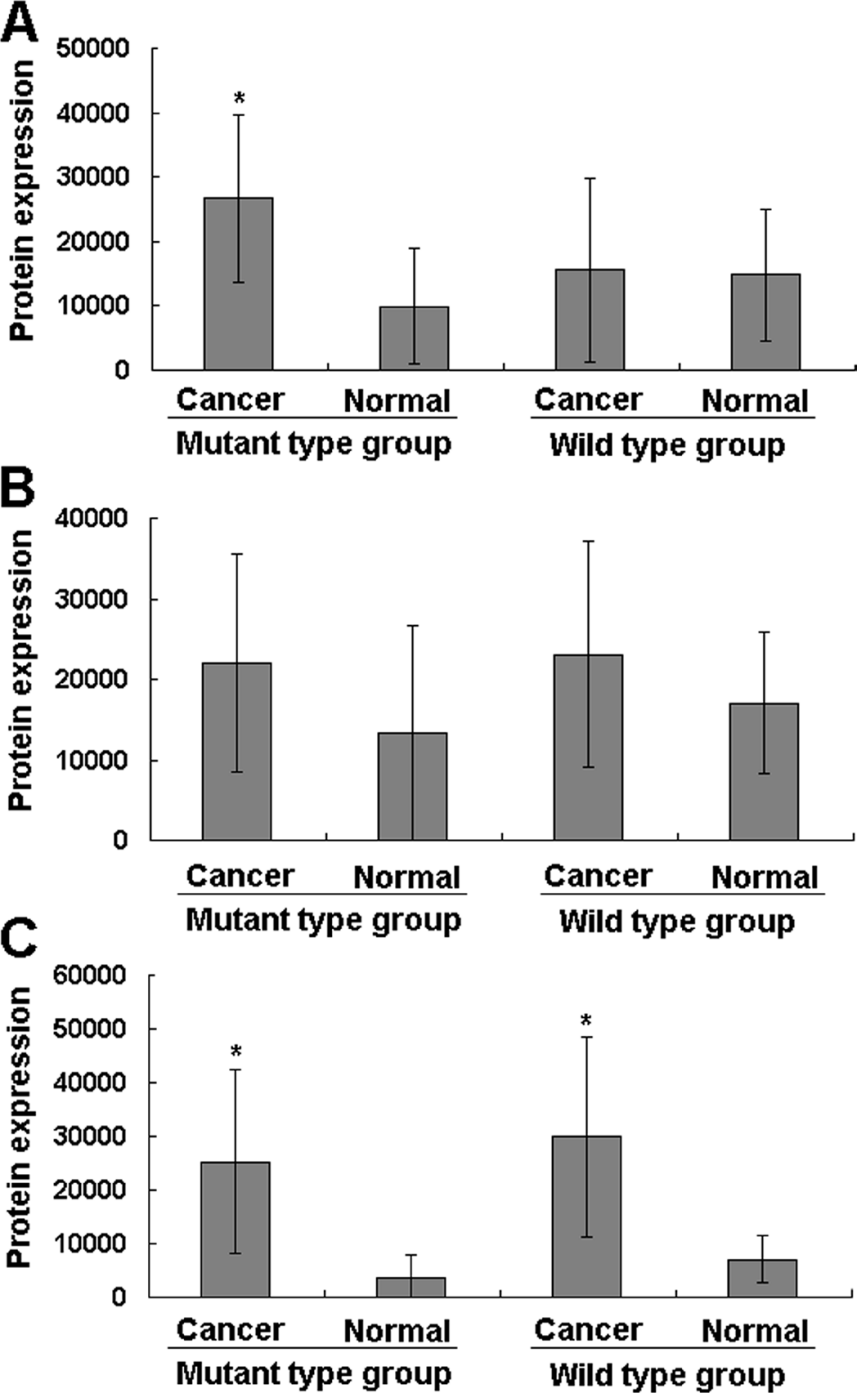
The statistics analyses of the β-catenin, C-myc and C reaction protein protein expression levels in the CRC patients (**A**) the expression level of the *C reaction protein* gene has statistical differences between the cancer and normal tissue in the mutant type groups, however there has no difference in the wild type group. The protein expression levels were normalized to GADPH. (**B**) There have no statistical differences of the *β-catenin* gene expression levels between cancer and normal tissues in all the mutant and wild type groups. (**C**) the expression levels of the *C-myc* gene in the cancer tissues were higher than the normal tissue in all the mutant and wild type groups.

We used the western blotting analysis to measure the expression levels of *CHIT1*, C reaction protein, C-myc and β-catenin genes in cancer and normal tissues in the wild and variant types of the gene. The expression levels of *CHIT1* were higher in cancer than in normal tissues in both heterozygous and homozygous variation type groups (Figure 4A, Figure 4B) but not in the wild type *CHIT1* group (Figure 4C, 4D). Like the *CHIT1* gene, the expression levels of *C reaction protein* gene also exhibited statistically significant differences between the cancer and normal tissues in the *CHIT1* gene mutant type groups (Figure 5 and Figure 6A). For the *β-catenin* gene, no statistical differences of expression levels were seen between the cancer and normal tissue in all the *CHIT1* gene mutant and wild type groups (Figure 5 and Figure 6B); the expression level of *C-myc* in all the *CHIT1* gene mutant and wild type groups had statistical differences between the cancer and normal tissues (Figure 5 and Figure 6C).

### Comparative analysis of clinical features

We also compared the clinical characteristics between wild type and variant *CHIT1* gene groups of the CRC subjects in detail. We found statistically significant differences between the two groups in several clinical features, such as hemoglobin, albumin, CEA, CA199, but not in others, such as gender composition, age, white blood cells, neutrophilic granulocyte percentage, creatinine, TNM Stage and histopathological types (Table 4).

**Table 4:** Comparative analysis of clinical features between mutant and wild type groups

Clinical Index	Wild Type	Mutant Type	Chi-Square Test
***Gender (male/female)***	148/99	48/25	*P* = 0.369
***Age (year)***	59.42 ± 12.65	58.89 ± 11.96	*P* = 0.554
***White Blood Cells***	6.79 ± 2.53	6.56 ± 2.10	*P* = 0.798
***Neutrophilic Granulocyte Percentage***	61.27 ± 11.31	58.89 ± 9.51	*P* = 0.059
***Hemoglobin***	128.68 ± 22.67	125.78 ± 28.06	*P* = 0.013
***Albumin***	40.40 ± 5.15	47.15 ± 46.02	*P* = 0.007
***Creatinine***	75.06 ± 16.82	78.96 ± 22.54	*P* = 0.100
***TNM Stage (I/II/III/IV)***	37/103/88/20	13/37/20/2	*P* = 0.184
***Histopathological types (TA/PA/MA/SC/AC/MC/CH/UC)***	51/11/6/2/0/1/0/1	163/44/19/7/1/4/1/9	*P* = 0.963
***CEA***	13.36 ± 44.40	29.72 ± 126.55	*P* = 0.002
***CA199***	59.71 ± 201.10	26.64 ± 67.68	*P* = 0.024
***Tumor sites (left/right)***	128/36	26/23	*P* = 0.001
***Tumor types (rectum/colon)***	134/108	32/39	*P* = 0.126

TA: Tubular adenocarcinoma; PA: Papillary adenocarcinoma; MA: Mucinous adenocarcinoma; SC: Signet-ring cell carcinoma; AC: Adenosquamous carcinoma; MC: Medullary caricinoma; CH: Choriocarinoma; UC: Undifferentiated caricinoma.

## DISCUSSION

In this study, we investigated the associations between *CHIT1* gene variants (rs61745299 and rs35920428) and the risk of colorectal cancer in the Chinese Han population. We found that two variations within the CDS region of *CHIT1* gene were associated with the risk of CRC. The expression levels of *CHIT1* and *C-reaction protein* genes were also associated with the variations.

Tens of trillions of microbes in the human distal intestine form the gut microbiota [19], which has different compositions among different human populations, associated with multiple factors such as long-term dietary habits of the subjects [20, 21]. The microbiota colonizing the mammalian gut can protect the host against pathologies [22]. Microbes and their metabolites not only facilitate human immune development [23] but also help prevent invasion by pathogens [24], playing very important roles for the human heath.

Some microbes in the microbiota may trigger intestinal inflammation [25]. Normally, the activated inflammatory cells along with other host responses will eliminate or kill the invading organisms [26]. However, in some cases the inflammatory responses may transform into chronic inflammations [27] or facilitate carcinogenesis [26, 28, 29]. Several inflammatory biomarkers, such as C-reactive protein, have been reported in the colorectal cancers process [30]. In this work, we characterized two variations rs61745299 and rs35920428 within the *CHIT1* gene that were associated with elevated expression levels of the C-reaction protein in the cancer tissue. This finding further proved the importance of the C-reactive protein in the pathogenesis of the disease. In addition to inflammation, the infectious agents are also involved in cancer development, especially in organs exposed to microorganisms, such as the colon and rectum [19, 31].

Chitotriose as a component of the bacterial cell wall renders the bacteria, especially some pathogenic bacteria, selective growth advantages [11, 12] and may enable them to promote inflammation processes [32]. The chitotriosidase encoded by the *CHIT1* gene is a highly conserved enzyme [33, 34] and promotes degradation of chitotriose and chitin-containing pathogens [33, 34]. In this work, we found the two variations associated with differential expression of *CHIT1* and risk of CRC, providing further evidence for the importance of the chitotriosidase in the pathogenesis of CRC.

Chitinase 3-like-1 (CHI3L1), another chitinase secreted by the colonic epithelial cells, contributes to the proliferation, migration, and neoplastic progression of colonic epithelial cells in the inflammatory conditions [35]. It is also a molecule associated with inflammation, and its expression is increased in colitis and colon cancer patients [36]. Therefore, it is interesting that we found the variations in the *CHIT1* gene associated with the increased expression of the *CHIT1* and *C-reaction protein* genes in the cancer tissue, further emphasizing the importance of chitotriosidase, chitotriose and microbiota in pathogenesis of colorectal cancer

The Wnt/β-catenin signaling pathway is highly conserved in evolution and found in all metazoan animals [37], and it regulates many life activities and processes [38–40]. Many cases of dysregulation in the pathway have been shown to promote colon tumorigenesis [41]. In the Wnt/β-catenin signaling pathway, the unbound β-catenin in cytoplasm may activate the Wnt cascade [5, 42]. Silencing of the β-catenin leads to decreased colonosphere formation [43] and hyper-activation of C-myc leads to increased potential formation of colonospheres [44]. However, in this work we did not find any differences of the expression levels of the *β-catenin* and *C-myc* genes between the *CHIT1* gene variant and wild type groups.

In conclusion, we found in this study that two variations rs61745299 and rs35920428 in the *CHIT1* gene were associated with the risk of CRC and expression levels of the *CHIT1* and *C-reaction protein* genes in the cancer tissue, providing further evidence for the key roles of chitotriose, chitotriosidase and C-reaction protein in the intestinal inflammation and CRC pathogenesis.

## MATERIALS AND METHODS

### Study population

In total, 320 sporadic adenomatous polyposis cases and 404 normal controls were collected for verification in this study (Table 1). All the subjects were assembled at the Department of Colorectal Surgery and Medical Examination Center of the Second Affiliated Hospital of Harbin Medical University, Harbin, China, and they all had physical and enteroscopic examinations. All patients had colorectal cancers and none of the normal controls showed enteric or other abnormality or defects. The medical history and physical characters of all the subjects were recorded in detail. We obtained a written informed consent from each participant or their guardian, and this work has been reviewed and approved by the Ethics Committee of Harbin Medical University [5], consistent with the 1975 Declaration of Helsinki.

### DNA analysis

Using standard protocols, we extracted the genomic DNA from peripheral blood leukocytes of the participants, and amplified the ten exons and splicing sites of the gene by polymerase chain reaction (PCR) using the primers shown in Table 5, and the PCR products were sequenced for mutational analysis [45].

**Table 5:** PCR primers used for CHIT1 gene sequence analysis

Exon	Forward primer	Reverse primer	Size (bp)	Tm (°C)
1	CTTCCCGCTTTCCTCTGT	TGGTAGCAAGTGGTCCCT	370	55.0
2	GCCTGGGAAGGTGAGAAT	AGGGCTGGTAGCAGATGG	248	54.2
3	**TGCCATTTCTGCCTGTCG**	**TGGGAGGAGGTTATCTGTC**	**411**	**54.6**
4	GATGGTCCCCTTTCCTCA	GCCCAGGTAGATGTTCACTT	536	56.4
5	CATCTACCTGGGCTCACA	CTGGAACAGGGCAGCAGT	423	53.8
6	GTCTGGGTCACCTTCTGC	CCATCAGCCAAGATGCTC	501	55.0
7	**GGGCTGGAATCCTAACAA**	**CAGAAACGGTGGGAGAAG**	**411**	**55.3**
8	CATGCCATCTTGAATTTATC	AAGGAGACTCACCCTTGA	421	52.7
9	TCTCCAGAATCTACAGCCACTC	GCAGGCATTGCTACAACC	481	55.1
10	TGTGAGGCCAGGTGTTGC	AGCCCAGGAGACCCAGAA	633	57.2

### Rs61745299 and rs35920428 *CHIT1* SNP genotyping analysis and statistical analysis

Gene variations were determined for 320 sporadic adenomatous polyposis cases and 404 normal controls to determine the genotypes (Table 5). The statistical analyses were conducted using the SPSS software (version 19.0), with those of the continuous variables (measurement data, such as age and protein expression level) by independent-samples *T* test and those for the discrete variables (enumeration data, such as gender composition and genotype frequency) by Chi-Square Tests to calculate odds ratios and *P* value [46]. *P* values less than 0.05 were considered statistically significant. The Hardy-Weinberg equilibrium test of the CRC and control population was conducted with the online software OEGE.

### Multiple sequence alignment

From the NCBI website (http://www.ncbi.nlm.nih.gov/), we obtained the CHIT1 protein sequences of various species and carried out multiple-sequence alignments of the proteins using the Vector NTI software.

### Western blotting analysis

Proteins of the tumor tissue and normal tissue near the tumor were extracted using standard protocols and protein contents were determined using the BCA protein assay kit (from BOSTER) and ELISA. Then the proteins were separated by 8% SDS-PAGE and transferred to PVDF membrane. The membranes then were incubated with the primary antibodies against CHIT1 (Abcam), C reaction protein (Abcam), C-myc (Abcam) and β-catenin (Abcam) and GAPDH (Abcam) in 5% non-fat milk in TBST at room temperature for two hours. After three washes, ten min each with TBST, the membranes were incubated with secondary antibodies (ZSGB-BIO) at room temperature for two hours. Then the membranes were developed using the enhanced chemiluminescence plus reagent and imaged using the Bio-Rad gel imaging system. The band value was read using the image J software.
